# Supplementary material for: Application of Plasma Activation in Flame-Retardant Treatment for Cotton Fabric
Source: Polymers (Basel). 2020 Jul 16;12(7):1575. doi: 10.3390/polym12071575 (PMC7408165; doi:10.3390/polym12071575)
Supplement: Supplementary file 1 [file polymers-12-01575-s001.pdf]

polymers

MDPI

*Article*

# Application of Plasma Activation in Flame-Retardant Treatment for Cotton Fabric

**Huong Nguyen Thi [1,2], Khanh Vu Thi Hong [1,*], Thanh Ngo Ha [1] and Duy-Nam Phan [1]**

[1] School of Textile – Leather and Fashion (STLF) - Hanoi University of Science and Technology (HUST), No. 1, Dai Co Viet, Hai Ba Trung, Hanoi, 100803, Viet Nam; huongnt87@hict.edu.vn (N.T.H); thanh.ngoha@hust.edu.vn (N.H.T); nam.phanduy@hust.edu.vn (P.D.N.)

[2] Hanoi Industrial Textile Garment University, Le Chi, Gia Lam, Hanoi, 100803, Viet Nam

* Correspondence: khanh.vuthihong@hust.edu.vn ; Tel.: +84-9034-463-18

Received: 04 June 2020; Accepted: 10 July 2020; Published: date

## Appendix

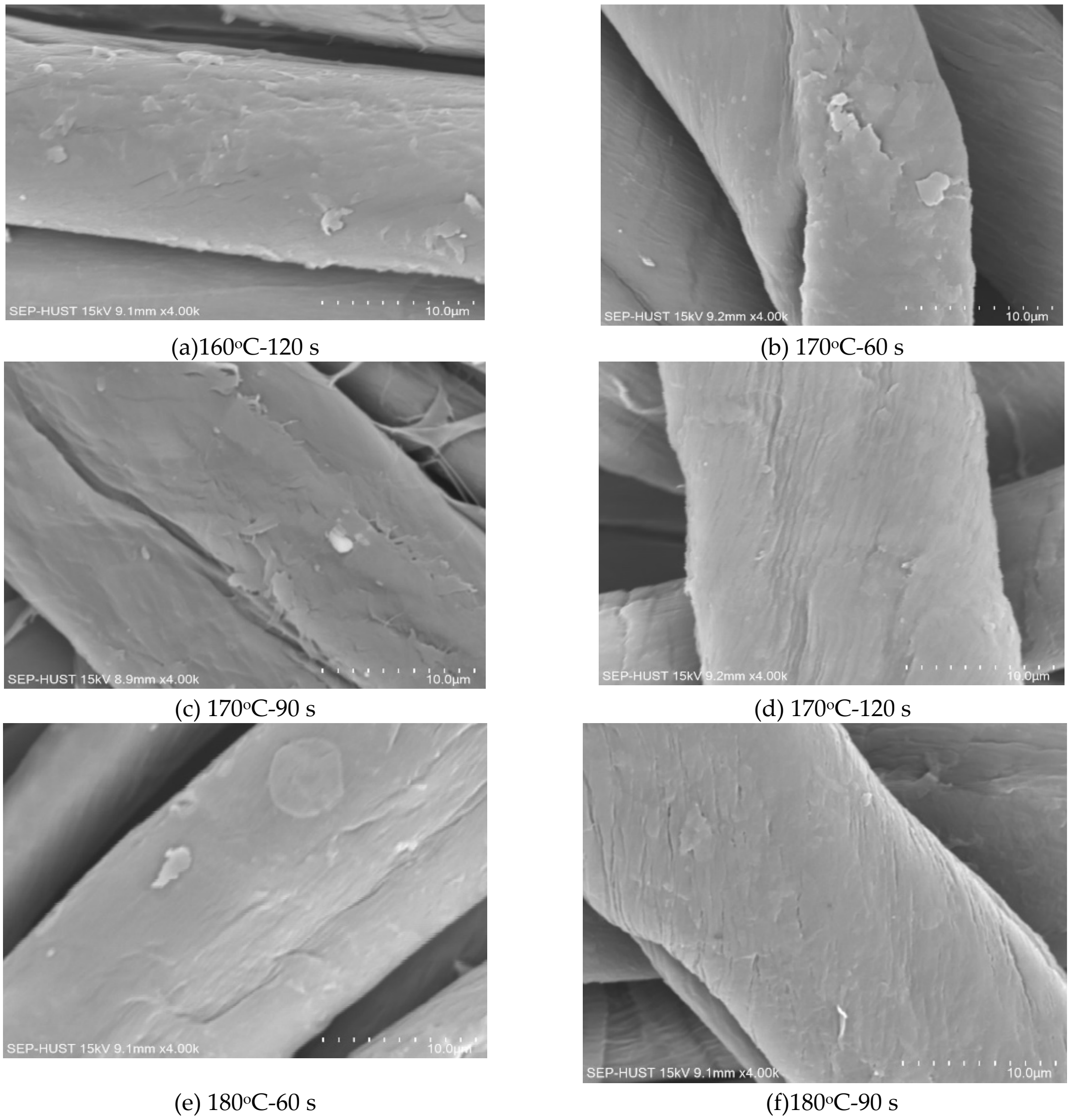


**Figure A1.** SEM images of the samples cured at different curing condition (curing temperature (°C) – curing time (s)) at a magnification of 4000×

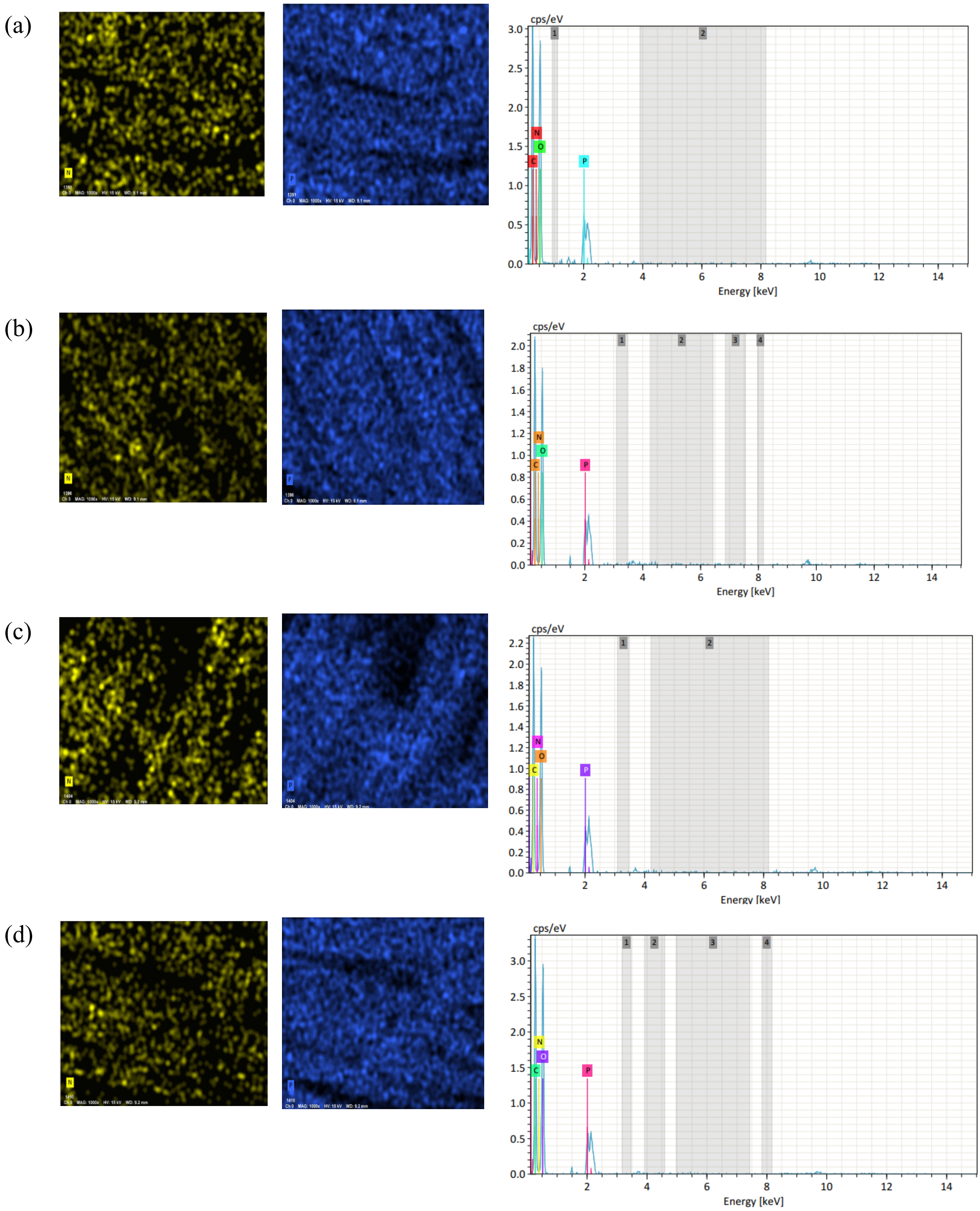
(a)
(b)
(c)
(d)
cps/eV
Energy [keV]
N
O
C
P
1
2
3
4

(e)

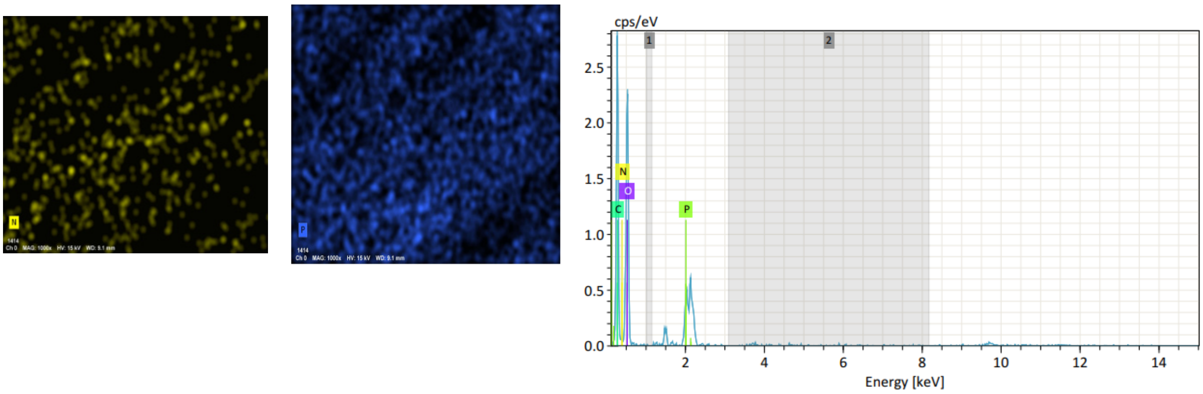


(f)

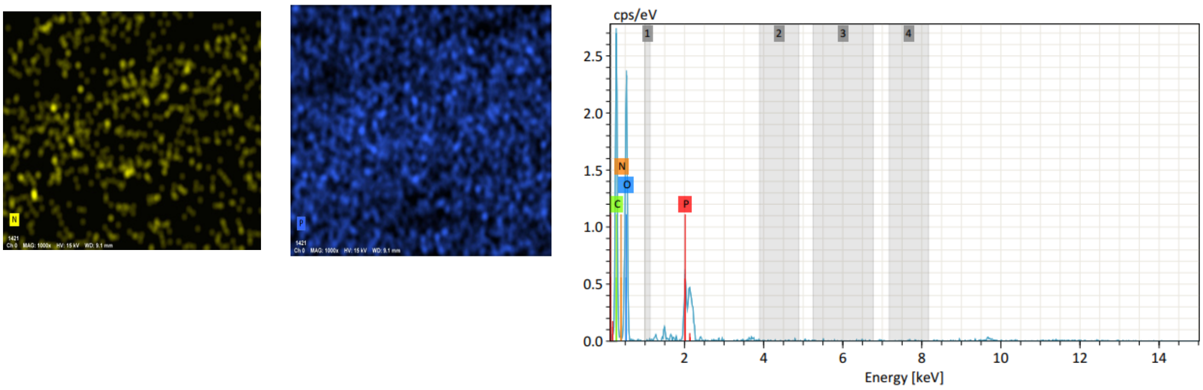


**Figure A2**. EDX N, P mapping and elemental spectral profile of (a) cured at 160°C for 120 s; (b) cured at 170°C for 60 s; (c) cured at 170°C for 90 s; (d) cured at 170°C for 120 s; (e) cured at 180°C for 60 s; (f) cured at 180°C for 90 s

© 2018 by the authors. Submitted for possible open access publication under the terms and conditions of the Creative Commons Attribution (CC BY) license (http://creativecommons.org/licenses/by/4.0/).
